# Salami-Tactics: when is it time for a major cut after multiple minor amputations?

**DOI:** 10.1007/s00402-021-04106-5

**Published:** 2021-08-09

**Authors:** Martin C. Berli, Zoran Rancic, Madlaina Schöni, Tobias Götschi, Pascal Schenk, Method Kabelitz, Thomas Böni, Felix W. A. Waibel

**Affiliations:** 1 Division of “Prosthetics and Orthotics”, Department of Orthopedics, Balgrist University Hospital, Forchstrasse 340, 8008 Zürich, Switzerland; 2 Clinic for Vascular Surgery, University Hospital Zurich, and Medical Faculty, University of Zurich, Zurich, Switzerland; 3 Department of Orthopaedic Surgery, University of Zurich, Institute for Biomechanics, ETH Zurich, Balgrist Campus, Zurich, Switzerland

**Keywords:** Major Amputation, Minor Amputation, PAD, Diabetes, Ulcer, Osteomyelitis

## Abstract

**Introduction:**

Repetitive minor amputations carry the concomitant risks of multiple surgical procedures, major amputations have physical and economical major drawbacks. The aim of this study was to evaluate whether there is a distinct number of minor amputations predicting a major amputation in the same leg and to determine risk factors for major amputation in multiple minor amputations.

**Materials and methods:**

A retrospective chart review including 429 patients with 534 index minor amputations between 07/1984 and 06/2019 was conducted. Patient demographics and clinical data including number and level of re-amputations were extracted from medical records and statistically analyzed.

**Results:**

290 legs (54.3%) had one or multiple re-amputations after index minor amputation. 89 (16.7%) legs needed major amputation during follow up. Major amputation was performed at a mean of 32.5 (range 0 – 275.2) months after index minor amputation. No particular re-amputation demonstrated statistically significant elevated odds ratio (a.) to be a major amputation compared to the preceding amputation and (b.) to lead to a major amputation at any point during follow up. Stepwise multivariate Cox regression analysis revealed minor re-amputation within 90 days (HR 3.8, 95% CI 2.0-7.3, *p* <0.001) as the only risk factor for major amputation if at least one re-amputation had to be performed.

**Conclusions:**

There is no distinct number of prior minor amputations in one leg that would justify a major amputation on its own. If a re-amputation has to be done, the timepoint needs to be considered as re-amputations within 90 days carry a fourfold risk for major amputation.

**Level of evidence:**

Retrospective comparative study (Level III).

**Supplementary Information:**

The online version contains supplementary material available at 10.1007/s00402-021-04106-5.

## Introduction

Minor foot amputations are common surgical interventions and feared consequences of complications in diabetic patients, patients with foot osteomyelitis, and patients suffering from peripheral arterial disease (PAD) [1–12]. Permanent disease (i.e. uncontrolled diabetes), or progression of atherosclerosis with consequent limb ischemia, often lead to more than one minor amputation [13–17]. A certain amount of these patients needs major amputation once the underlying conditions of the feet are not manageable anymore. In these multimorbid patients, each surgical intervention carries a substantial risk of perioperative morbidity, and therefore should be avoided or limited to a minimum, which in summary would favor an early major amputation [18–22]. However, major amputations lead to an increased oxygen consumption and cardiac effort, change the mobility level of the patient and necessitate auxiliary means [23–28].

Currently, the decision on whether to perform multiple minor amputations or an early major amputation strongly varies between countries and surgeons, but there is a trend in favor of performing multiple minor amputations [29, 30]. Consensus exists only about the importance of vascular perfusion when determining the amputation level [31–33]. Therefore, the decision to perform either multiple minor or one major amputation remains up to the surgeon’s expertise. Health instruments guiding the surgeon`s suggestion are missing [34].

Thus, the primary goal of this study was to investigate whether there is a threshold number of minor amputations that result in a major amputation. Secondary goal of the study was to identify risk factors for primary, and each other minor amputation, and to determine risk factors for major amputation in case of ≥ 2 minor amputations performed at the same foot.

## Materials and methods

**Fig. 1:**
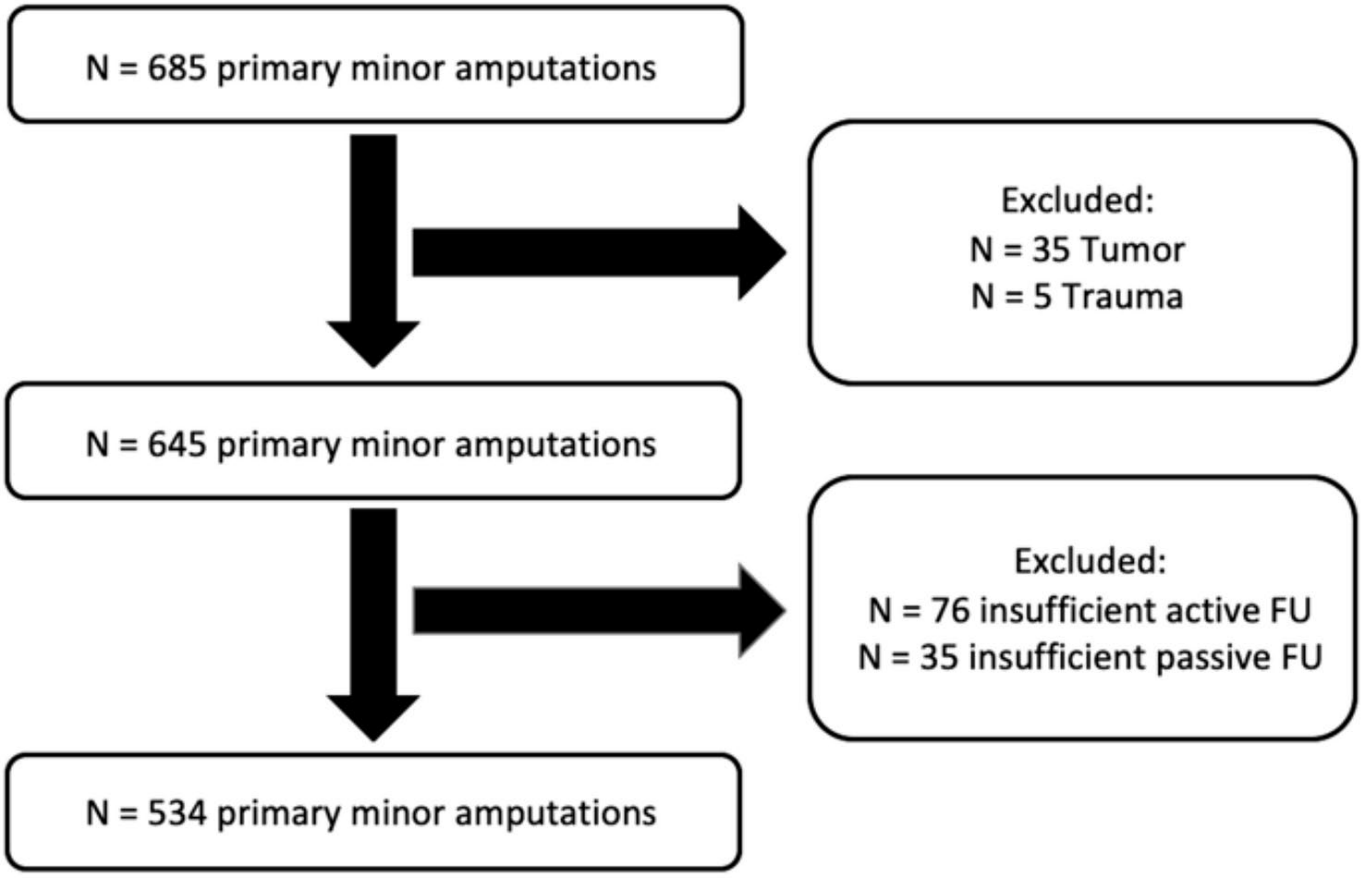
Flowchart demonstrating patient inclusion and exclusion

We conducted a retrospective chart review of prospectively collected data of all consecutive minor lower extremity amputations performed at our institution, which is specialized in the treatment of diabetic and dysvascular patients—between July 1^st^ 1984 and November 30th, 2018. Inclusion criteria were first time minor amputation of the affected lower extremity, a possible passive follow up of at least one year and an active follow up of at least 90 days after index minor amputation. We refrained to exclude patients who died within 90 days after the index minor amputation and received major amputation prior to death. Amputations were considered “minor” until and including the Syme level and “major” starting with the transtibial level or more proximal. Exclusion criteria were prior amputation procedures at the same foot, amputations due to trauma, and malformations or tumor. A flowchart containing inclusion and exclusion details is given in Fig. 1. This study was approved by the local research ethics committee (BASEC number 2016–000387). Informed consent was obtained appropriate to local research ethics committee regulation.

Patient demographics and clinical data were derived from the institutional electronic medical records and are shown in Table 1. All amputations were performed by or under the direct supervision of one of two certified attending orthopedic surgeons (MCB or TB) with more than 15 years of surgical experience. When Osteomyelitis was clinically suspected (wound persisting > 3 months, bone visible within open wound, positive probe to bone test), it was confirmed by a combination of radiographs and MRI or nuclear medicine techniques (the latter when MRI was impossible due to implants such as pacemakers). The extent of osteomyelitis and thereby the amount of bone to be resected was determined by the amount of loss of focal signal in T1 sequences [35]. Information collected included patient demographic data, medical history and surgical details of amputations. Peripheral arterial disease was graded according to the Fontaine classification in stages 1 to 4 by angiologists [36]. The Fontaine classification is based on the clinical presentation of peripheral arterial disease (PAD) and contains four stages: stage one is asymptomatic PAD, stage two demonstrates mild claudication (IIA: claudication at a distance > 200 m, IIB: claudication at a distance < 200 m), stage three pain at rest while stage four demonstrates necrosis and/or gangrene [36]. Follow-up visits were scheduled according to a standardized scheme: the first visit was performed one week after hospital discharge. Further visits were scheduled every 7–10 days as long as the surgical wound or plantar ulcers were not healed and until transfer from the postoperative off-loading device (e.g. therapy shoe or cast) to a definitive foot protecting device (ranging from orthopedic insoles to orthopedic shoewear) had been realized. Upon verification of wound and/or ulcer healing, the next visit was scheduled 4–6 six weeks later. Given there were no skin lesions at this 4–6 six weeks visit, the next visits were scheduled every three months. Visits occurred either in our hospital`s outpatient clinic or in our wound care nursery.

**Table 1:** Patient demographics

Characteristic	Value	
Age, years, mean (range)	67.3 (19 – 96)	
Sex, individuals (%)	f: 110 (25.6%)	m: 319 (74.4%)
Sex, feet (%)	f: 133 (24.9%)	m: 401 (75.1%)
Side of operation	r: 177	bilateral: 105
	l: 147	
BMI, kg/m^2^, mean (range)	28.6 (16.0 – 57.0)	
FU, months, mean (range)	49 (0 – 279)	
	Yes: Number (%)	No: Number (%)
Diabetes mellitus (%)	430 (78.8%)	113 (21.2%)
Type I (% of DM)	115 (26.7%)	
Type II (% of DM)	305 (70.9%)	
Insulin treatment (% of DM)	304 (70.7%)	
HbA1c (%), mean (range)	8.1 (3.8 – 14.8)	
Peripheral neuropathy (%)	375 (70.2%)	159 (29.8%)
Smoking (%)	256 (47.9%)	278 (52.1%)
Pack years, years, mean (range)	38.2 (1–120)	
Alcohol abuse (%)	195 (36.5%)	339 (63.5%)
Chronic kidney disease (%)	272 (50.9%)	262 (49.1%)
Dialysis (%)	36 (6.7%)	498 (93.3%)
Peripheral vascular disease (%)	432 (79.2%)	102 (20.8%)
Fontaine classification: stage I (% of PAD)	96 (22.2%)	
Fontaine classification: stage II (% of PAD)	167 (38.6%)	
Fontaine classification: stage III (% of PAD)	20 (4.6%)	
Fontaine classification: stage IV (% of PAD)	140 (32.4%)	
History of PTA (%)	301 (56.4%)	233 (43.6%)

Statistical analysis was conducted with SPSS statistical software (IBM Corp. Released 2016. IBM SPSS Statistics for Windows, Version 24.0. Armonk, NY: IBM Corp.) Differences between groups were checked using students t-test for continuous demographic variables and using Chi-square test categorical demographic variables. The association between the consecutive number of surgery and the extent of the respective amputation was investigated using Chi-square tests. Odds ratios were calculated to compare the percentage of major amputations in rising numbers of re-amputations and to compare the percentage of major amputation during the further follow-up depending on the number of necessary minor re-amputations.

Kaplan–Meier survival estimates were calculated for major amputation free survival. The time of major amputation was selected as the primary endpoint. Following events were used as censor dates: death without major amputation; the last confirmed date without major amputation if the patient was lost to follow-up; date of data collection for this study, if no major amputation was done until this moment. Additionally, separate survival curves were plotted for different potential risk factors. Different survival curves were compared with the log-rank test.

In addition, the Hazard Ratio for major amputation was calculated for different patient groups using stepwise multivariate Cox regression analysis. Significance level was set to 0.05 for p-levels.

## Results

### Patient characteristics

There were 429 patients—319 men and 110 women – with 534 legs included. Mean follow-up was 49 (range 0 – 279) months. 91 (21.2%) patients died during follow-up at a mean of 63 (range 3 to 209) months after index minor amputation. Four patients died after having major amputation within 90 days.

### Minor and major re-amputations

In all 534 legs of our study population, a minor amputation was performed as the index procedure. 290 (54.3%) legs had one or more re-amputation. 89 (16.7%) legs had to undergo major amputation during follow up. Of the 89 major amputations, 47 major amputations were performed as the first revision after index minor amputations. 42 major amputations were performed after one or several minor re-amputations. Major amputation was performed after a mean of 32.5 (range 0 – 275) months after index minor amputation.

129/290 (44.5%) re-amputation cases needed their re-amputation within the first 90 days after index minor amputation; 98 cases (33.8%) needed a minor re-amputation, 20 cases (6.9%) a major amputation and 11 (3.8%) cases both a minor and a major amputation.

**Fig. 2:**
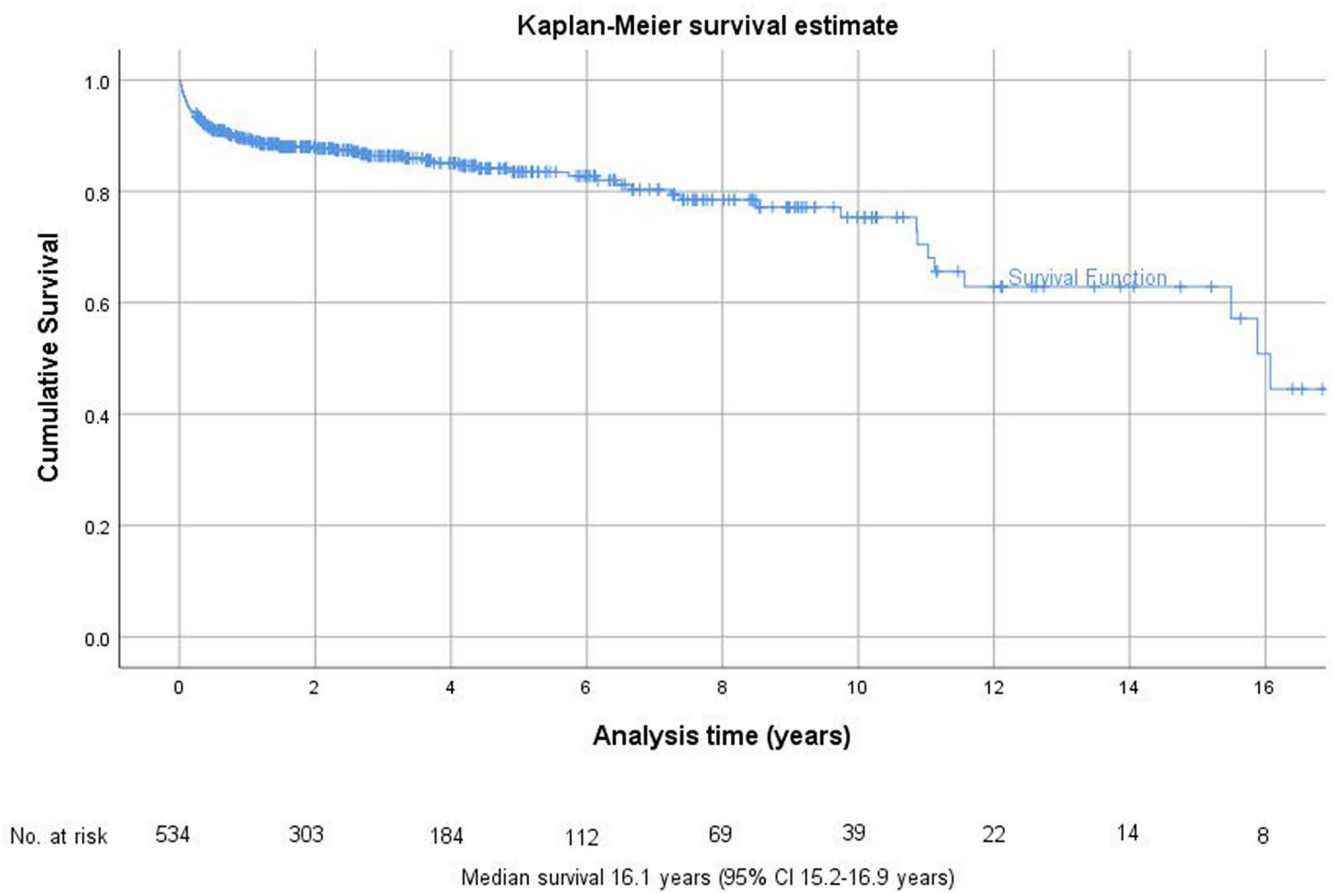
Kaplan–Meier analysis survivorship curve for major amputation free survival overall
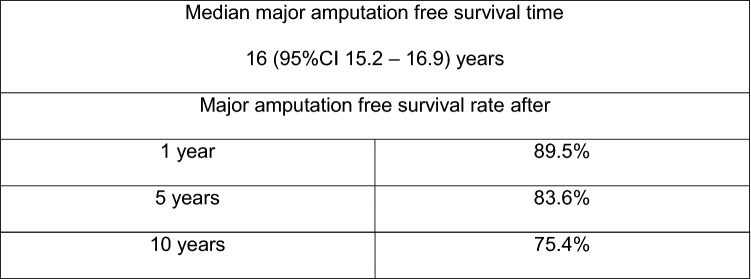

Major amputation free survival rate was 89.4% after 1 year (SD 1.4%; Number at risk 405), 83.5% after 5 years (SD 1.9%, Number at risk 132) and 75.3% after 10 years (SD 3.4%, Number at risk 39) (Fig. 2). The median major amputation free survival time was 16.1 years (95%CI 15.2 – 16.9 years).

Table 2 summarizes the results stratified by comorbidities diabetes mellitus and PAD.

**Table 2:** Results stratified pairwise by presence of comorbidities Diabetes mellitus and PAD (Diabetes: "yes" vs. "no"; PAD Fontaine stages 0–2 vs. 3–4; subanalysis of patients with PAD stages 3–4 with and without Diabetes), sorted by the frequency of major amputation

	Diabetes mellitus (*n* = 421)	No Diabetes mellitus (*n* = 113)	PAD stage 0 – 2 (*n* = 373)	PAD stage 3–4 (*n* = 160)	Diabetes mellitus + PAD stage 3–4 (*n* = 109)	No Diabetes mellitus + PAD stage 3–4 (*n* = 51)
Number of revisions (mean (SD))	1.0 (1.3)	0.9 (1.0)	1.0 (1.3)	0.9 (1.0)	1.0 (1.1)	0.7 (0.8)
Revision rate overall (%)	54.6%	53.1%	53.7%	55.6%	57.8%	51.0%
Time to first revision (mean, months)	18	18	20	12	11	13
Number of minor amputations until major amputation (mean (SD))	2.0 (1.4)	1.6 (0.9)	2.3 (1.6)	1.5 (0.8)	1.6 (0.9)	1.4 (0.5)
*Major amputation rate (%)*	*16.2%*	*18.6%*	*12%*	*27.5%*	*24.8%*	*33.3%*
Time to major amputation (mean, months)	33	31	50	14	14	15
No revision within 90 days	76.2%	74.3%	79.1%	68.1%	67.0%	70.6%
Minor amputation within 90 days	19%	15.9%	18.2%	18.8%	22.9%	9.8%
Minor + major amputation within 90 days	1.2%	5.3%	0.8%	5%	1.8%	11.8%
Directly major amputation within 90 days	3.6%	4.4%	1.9%	8.1%	8.3%	7.8%
Follow-up (mean, months)	51	42	53	41	44	34

### Influence of timepoint and number of minor re-amputations on major amputation risk

Most of the patients had one or two re-amputations (Table 3). We first compared re-amputations pairwise to determine the odds ratio of the next re-amputation to be a major amputation: there was no re-amputation step that had a significantly elevated odds ratio to be a major amputation compared to the preceding one. Subsequently we calculated the odds ratio for each re-amputation step to suffer a major amputation at any point during further follow up. Again, all calculated odds ratios were insignificant (Table 3).

**Table 3:** Amputation types depending on the increasing numbers of revision

Intervention	Total	Type of intervention		Odds ratio to be a major amputation		Major amputation during further FU	Odds ratio suffering a major amputation during further FU	
		Minor amputation	Major amputation	OR (95% CI)	*p*-value		OR (95% CI)	*p*-value
Index procedure	534	534	none	–	–	89	–	–
Revision 1	290	243 (83.8%)	47 (16.2%)	–	–	42	1.0 (0.7 – 1.6)	n.s
Revision 2 (vs. Revision 1)	135	113 (83.7%)	22 (16.3%)	1.0 (0.6 – 1.8)	n.s	20	1.0 (0.6 – 1.9)	n.s
Revision 3 (vs. Revision 2)	62	53 (85.5%)	9 (14.5%)	0.9 (0.4 – 2.0)	n.s	11	1.2 (0.5 – 2.8)	n.s
Revision 4 (vs. Revision 3)	27	21 (77.8%)	6 (22.2%)	1.7 (0.5 – 5.3)	n.s	5	1.2 (0.4 – 4.0)	n.s
Revision 5 (vs. Revision 4)	8	6 (75%)	2 (25%)	1.2 (0.2 – 7.3)	n.s	3	3.2 (0.5 – 21.2)	n.s
Revision 6 (vs. Revision 5)	4	2 (50%)	2 (50%)	3.0 (0.2 – 37.7)	n.s	1	1.0 (0.04 – 24.5)	n.s
Revision 7 (vs. Revision 6)	1	0 (0%)	1 (100%)	3.0 (0.1 – 115.3)	n.s	0	–	–

n.s. = not significant

Odds ratio was calculated for each re-amputation step to be a major amputation, compared to the previous revision (column "Odds ratio to be a major amputation"). Further, odds ratio was calculated for each re-amputation step to suffer a major amputation at any point over the follow up (column "Odds ration suffering a major amputation during further FU")

**Fig. 3:**
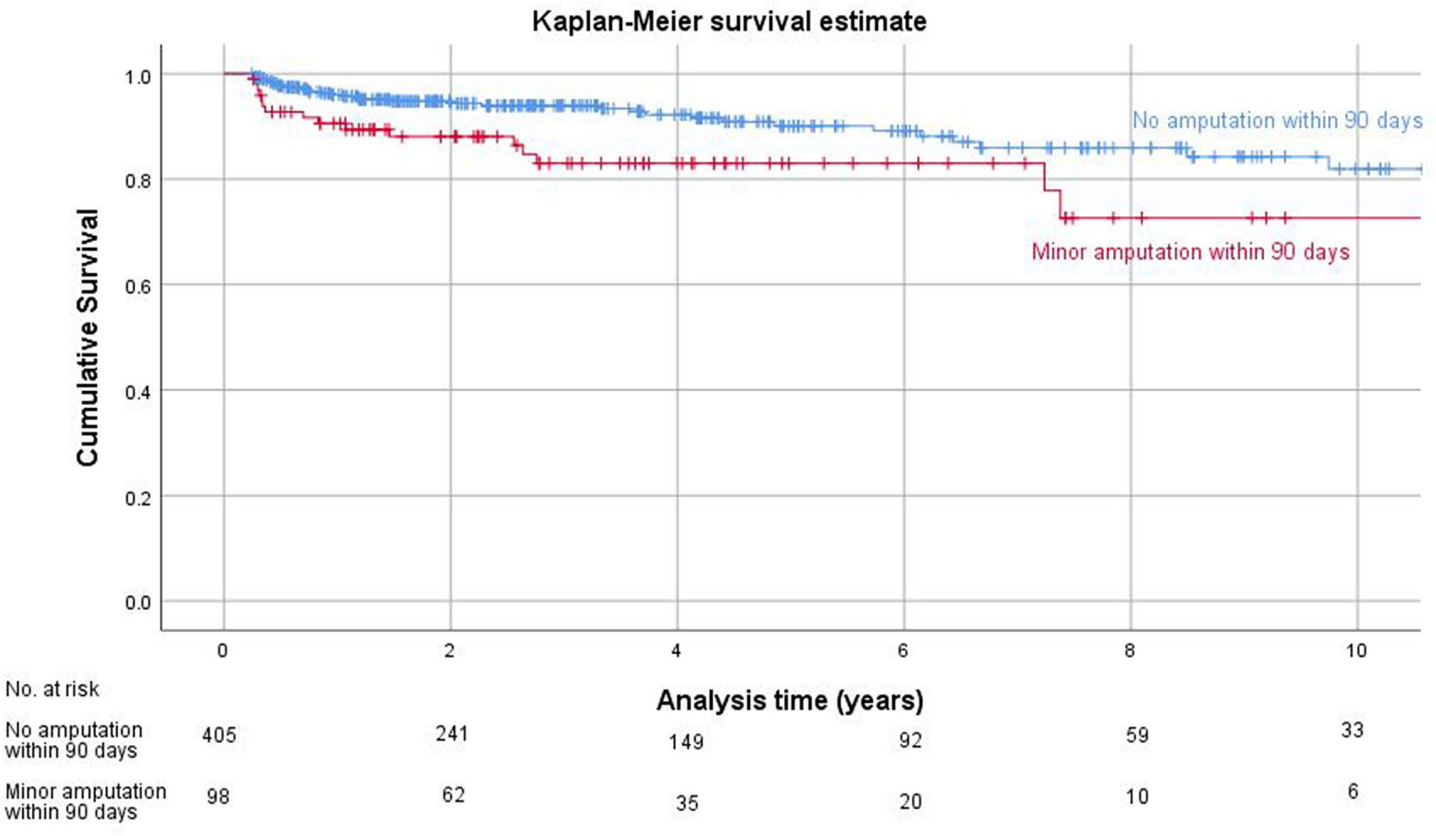
Kaplan–Meier analysis survivorship curve for major amputation free survival, minor amputation within 90 days "yes" vs. “no”
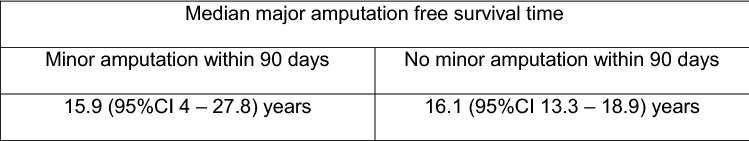

In cases without major amputation within the first 90 days after index amputation, patients without any re-amputation procedure within 90 days had a significant better major amputation free survival than patients that needed an early minor re-amputation (Log-Rank Test p = 0.04) (Fig. 3).

**Fig. 4:**
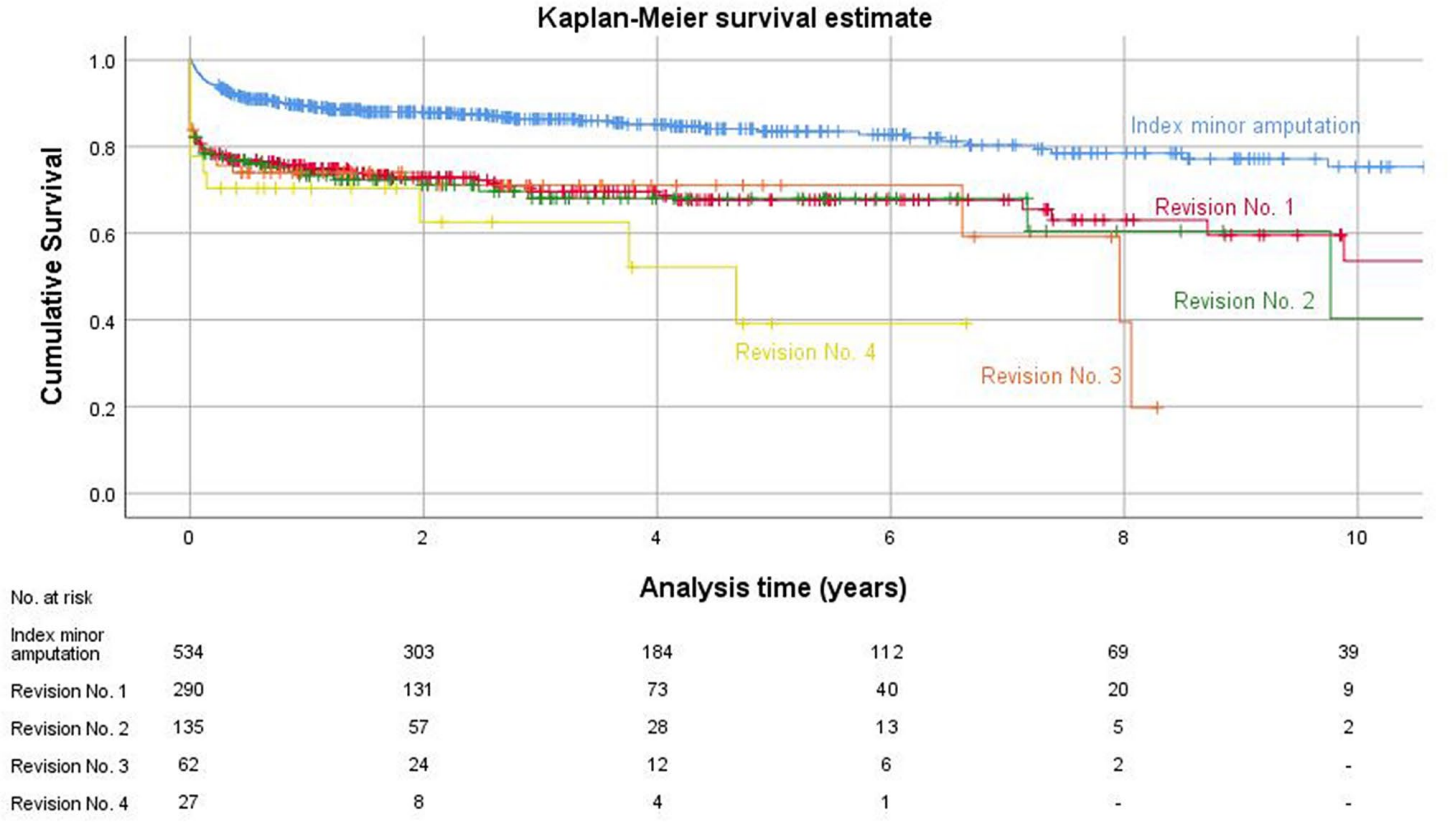
Kaplan–Meier analysis survivorship curve for major amputation free survival, for each surgical procedure
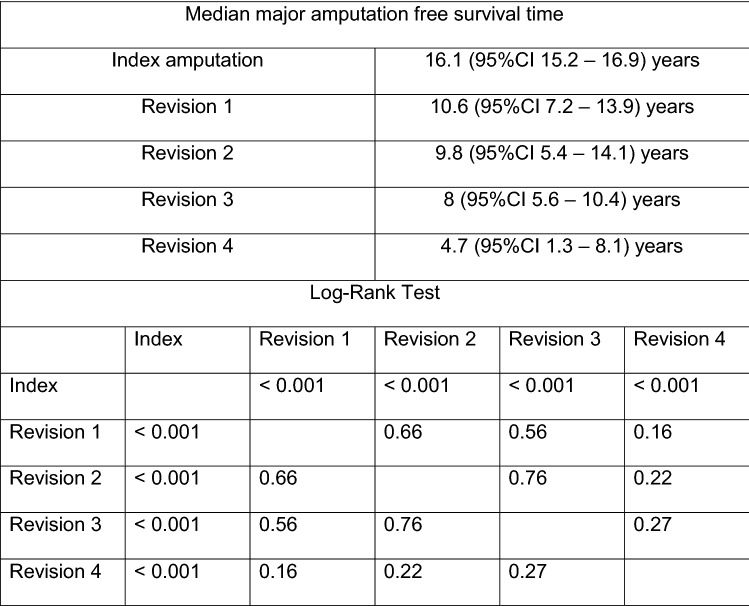

Kaplan–Meier survival estimates were performed for major amputation free survival, separated for each operation (index minor amputation, revisions 1 to 4; revision 5–7 were left out due to the limited number of cases). Revisions 1 to 4 demonstrated no statistical significance (Fig. 4, Log-Rank Tests given in Figure caption).

### Risk factors for major amputation

Stepwise multivariate Cox regression analysis revealed peripheral arterial disease stage 3–4 (HR 2.3, 95% CI 1.5—3.6, p < 0.001) and minor re-amputation within 90 days (HR 1.6, 95% CI 1.0–2.6, p = 0.032) as risk factors for major amputation. Osteomyelitis was negatively associated with major amputation (Cox regression: HR 0.6, 95% CI 0.4 – 0.9, p = 0.012).

Investigating only those patients with multiple minor amputations, stepwise multivariate Cox regression analysis revealed minor amputation within 90 days after index amputation as the only significant risk factor for major amputation (HR 3.8, 95% CI 2.0–7.3, p < 0.001).

**Fig. 5:**
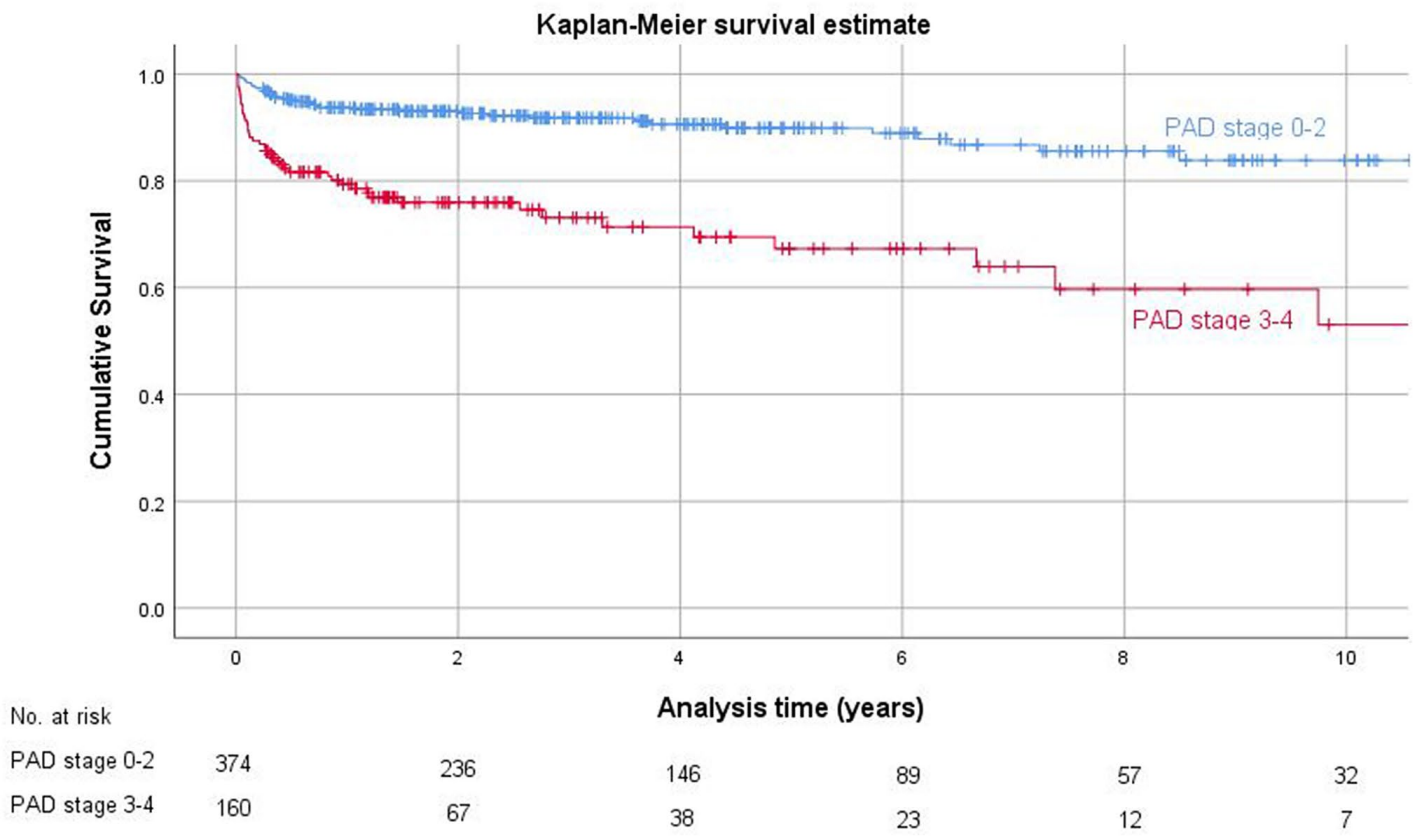
Kaplan–Meier analysis survivorship curve for major amputation free survival, "PAD stage 0–2" vs. "PAD stage 3–4"
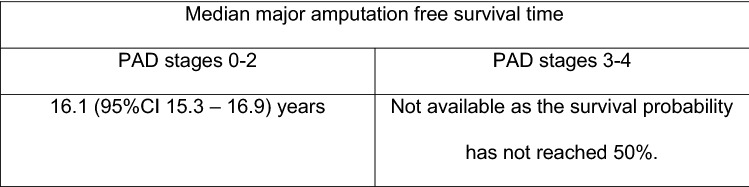

**Fig. 6:**
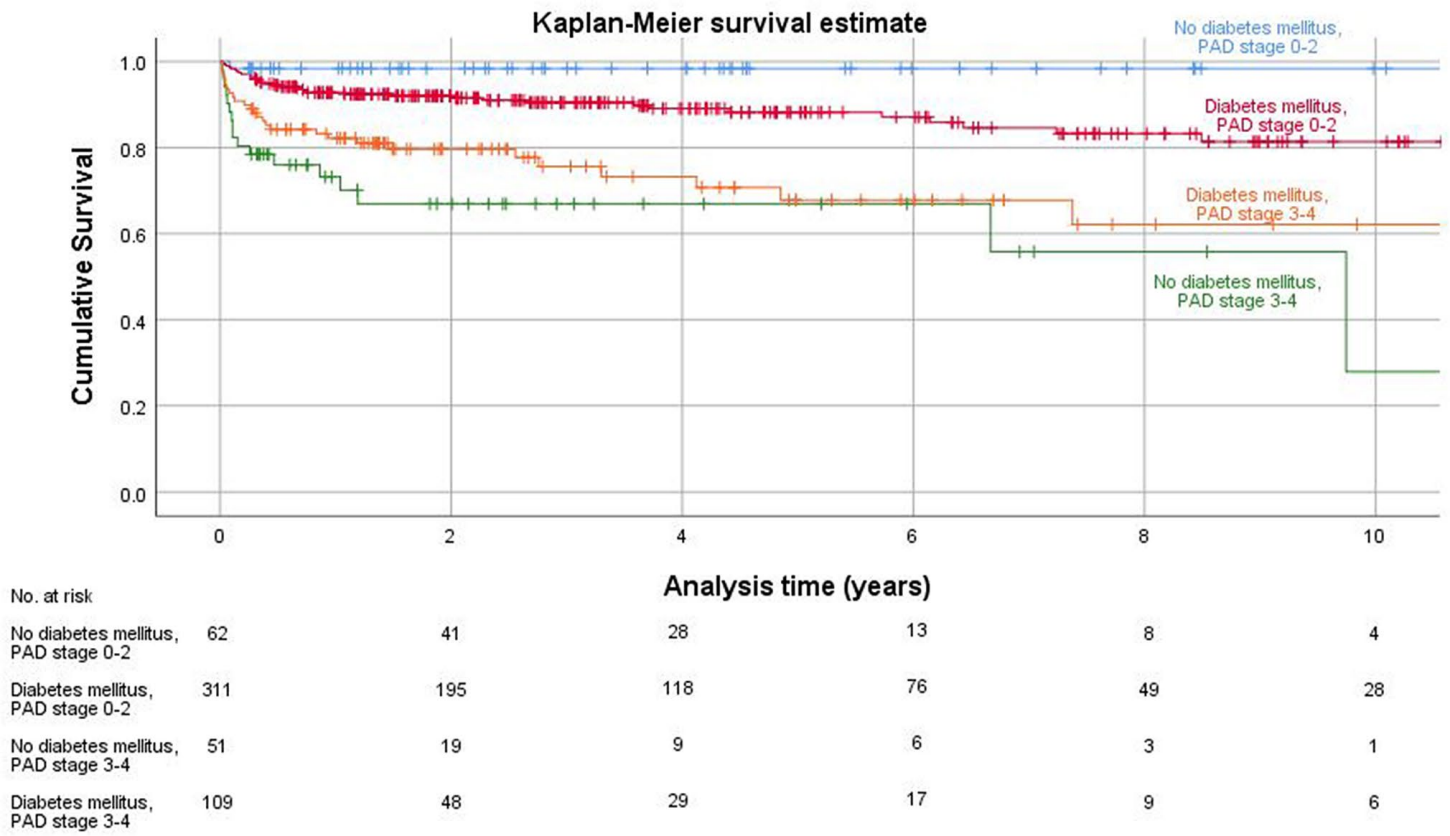
Kaplan–Meier analysis survivorship curve for major amputation free survival, combination of risk factors Diabetes mellitus and PAD stage
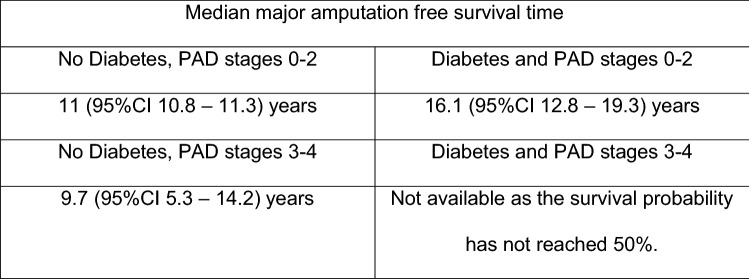

Kaplan–Meier survival curves were estimated comparing major amputation free survival in patients with PAD stages 1–2 versus 3–4 (Fig. 5), and combining presence of diabetes and PAD (Fig. 6). Results of the Cox regression analysis were confirmed.

## Discussion

The primary goal of this study was to investigate whether there is a threshold of minor amputations, that increases the probability of a future major amputation. No minor amputation step had statistically significant increased odds to be followed by a major amputation as the next step. Further, no minor amputation step demonstrated increased odds to have a major amputation as any subsequent amputation step. Hence, we could not identify a clear threshold of minor amputations that constantly leads to a major amputation. The only variable that was associated with major amputation in case of multiple minor amputations was minor revision surgery within 90 days of the index minor amputation.

Amputations are a feared consequence of complications in long-term diabetes mellitus and PAD [1–5]. Many of the concerned patients have to undergo more than one minor or even a major amputation [13–17]. Due to the numerous and significant comorbidities of these patients, each amputation contains a major risk for severe cardiac, renal, or other complications even if minor amputations can be performed in regional anaesthesia [18–22]. To date, there is no evidence leaning towards multiple minor versus one major amputation. Our study supports those who advocate multiple minor amputations. Neither PAD nor Diabetes were identified as independent risk factors for major amputation in case of multiple minor amputations. This allows surgeons to apply our results on both patient collectives.

PAD is a known risk factor for lower extremity amputations. Major amputation has declined over the last decade but is still performed for 7% of patients with peripheral artery disease over the course of their lifetime [37–41]. Nerone et al. compared patients with at least 1 subsequent minor amputation with patients with at least 1 subsequent major amputation after an initial minor lower extremity amputation in diabetic patients [42]. He found that the prevalence of major amputation after initial minor pedal amputation was statistically significantly associated with the presence of peripheral arterial disease. Gurney et al. found in a national cohort of people with diabetes that peripheral vascular disease conferred the greatest independent risk for major amputation (adjusted HR of 12.72) of all tested comorbid conditions [43]. We were able to confirm severe PAD stages as a risk factor for major amputation after initial minor amputation and were able to show that this is not only true for diabetic patients but for overall patients undergoing minor amputation. However, analyzing patients with multiple minor amputations only, PAD stages 3 and 4 lost their status as a risk factor for major amputation. We consider this finding logical and assume that it has been partially influenced by a bias of indication. In severe PAD stages, loss of tissue can make limb preservation impossible and therefore exclude the possibility of multiple minor amputations.

Griffin described a significant correlation between patients who did not have diabetes and future limb loss after toe amputation [44]. We could not confirm this effect, but diabetes had no impact on major amputation free survival in our study. This is consistent with the results of Sheahan et al., who reported that diabetes does not have an impact on limb salvage in their series of 920 episodes with minor amputations [45].

The seemingly protective effect of osteomyelitis on major amputation free survival is an interesting observation of our study. We attribute this finding to better vascular patency and to a lengthy antibiotic treatment in most of the osteomyelitis cases. Our results demonstrate that Osteomyelitis in diabetic patients with PAD stage 1–2 seems to be more benign than in diabetic patients with PAD stage 3–4 and gangrene.

Minor amputation within 90 days after index amputation was the single independent risk factor we could identify for major amputation in case of multiple minor amputations. Interestingly, the proportion of patients that needed revision minor amputation within 90 days was similar among patients with Diabetes (19%) and PAD (18%). Patients without Diabetes were less affected by early revision minor amputation (16%). We assume that presence of microangiopathy played a distinct role in early revision. While we did not investigate presence of microangiopathy this remains an assumption. As surgeons could be tempted to perform major amputation in this "revision within 90 days" scenario, the authors want to emphasize the importance of repeated vascular work-up in those cases before any irreversible decision is made.

In the authors opinion, the synthesis of the results of the present study is that multiple minor amputations at the same foot are worthwhile to be considered, depending on key comorbidities (first and foremost PAD stages 3 and 4) and on the chronological sequence after the index amputation.

Strengths of the study are the length of follow-up and the comparatively high number of patients. Also, the study reflects the expertise of two surgeons with over 15 years of experience each. However, the study has several limitations: First, it is a retrospective study design of prospectively collected data. Both collection and treatment bias would be conceivable. Second, despite the comparatively high number of patients, the numbers in individual groups were smaller so other influencing factors may not have been identified as significant. Third, the vascular evaluation before minor amputation, from the historical point of view, differs among the patients at the begin of the study and nowadays. Historically the complete vascular assessment was performed only in patients with PAD. In last 10 years vascular assessment was part of investigation before the minor amputation (ABI, or ultrasound examination followed by CTA, or angiography if the vascular perfusion was not adequate).

The authors conclude that there is no distinct number of prior minor amputations in one leg that would justify a major amputation on its own. The only variable that is associated with major amputation in case of multiple minor amputations is a minor amputation within 90 days after index amputation. It carries a fourfold risk of major amputation. The time point of re-amputation necessity must be considered when choosing the appropriate amputation level.

## Supplementary Information

Below is the link to the electronic supplementary material.

Supplementary file1 (DOCX 492 KB)

## Declarations

### Location of work statement

The study was conducted at Balgrist University Hospital, Zurich, Switzerland.
