# Supplementary figures and images for: Salami-Tactics: when is it time for a major cut after multiple minor amputations?
Source: Arch Orthop Trauma Surg. 2021 Aug 9;143(2):645–56. doi: 10.1007/s00402-021-04106-5 (PMC9925494; doi:10.1007/s00402-021-04106-5)

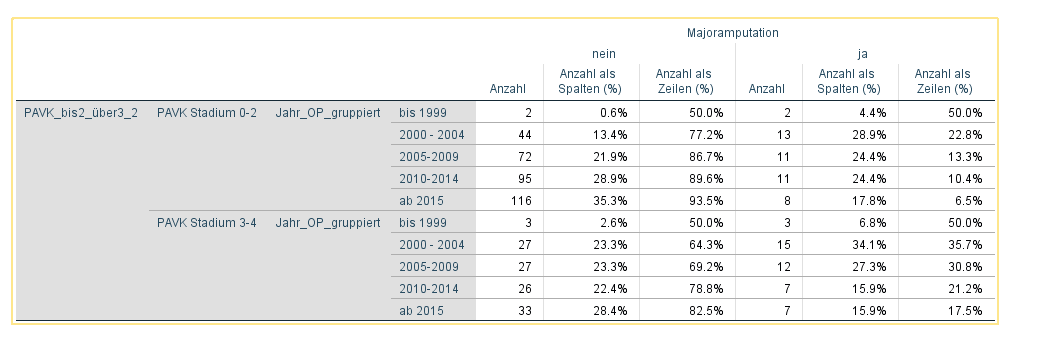


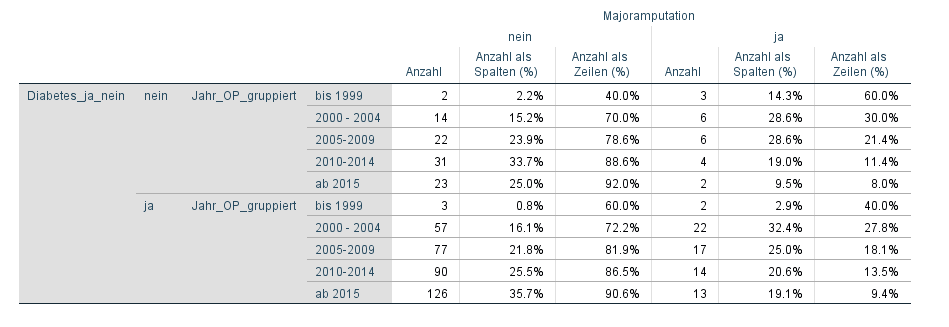


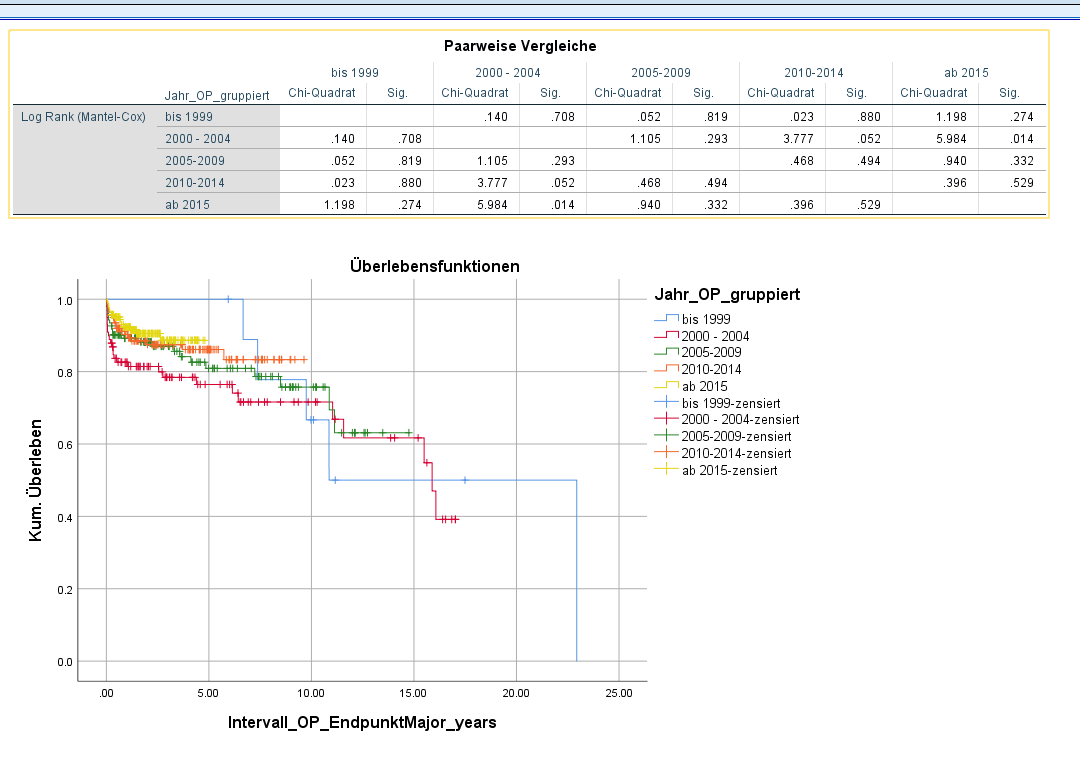


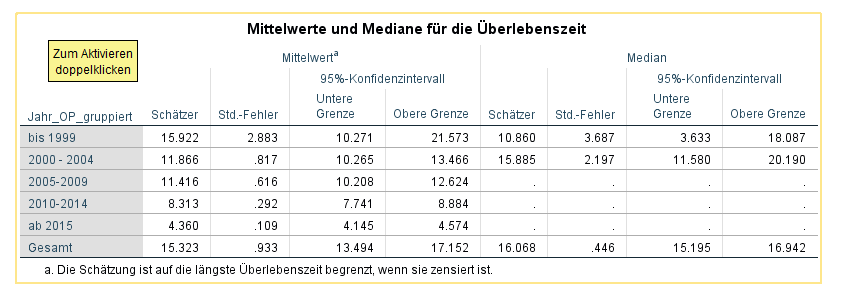


Overall Jahrzehnt


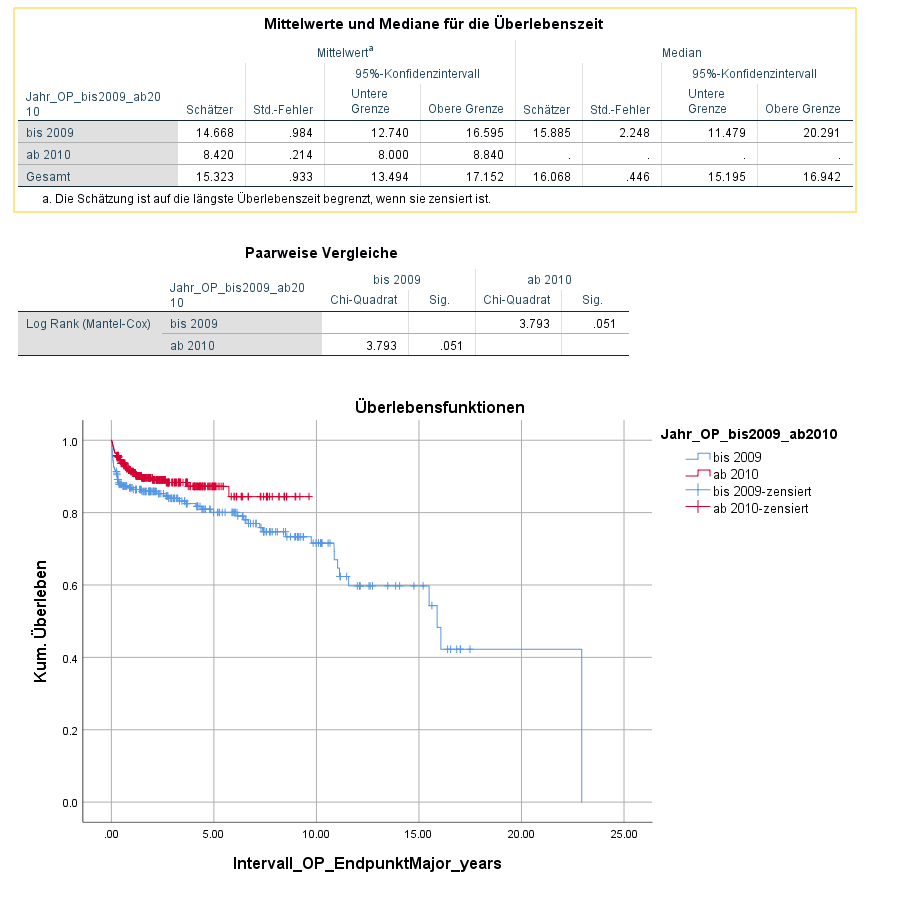


Diabetes NO


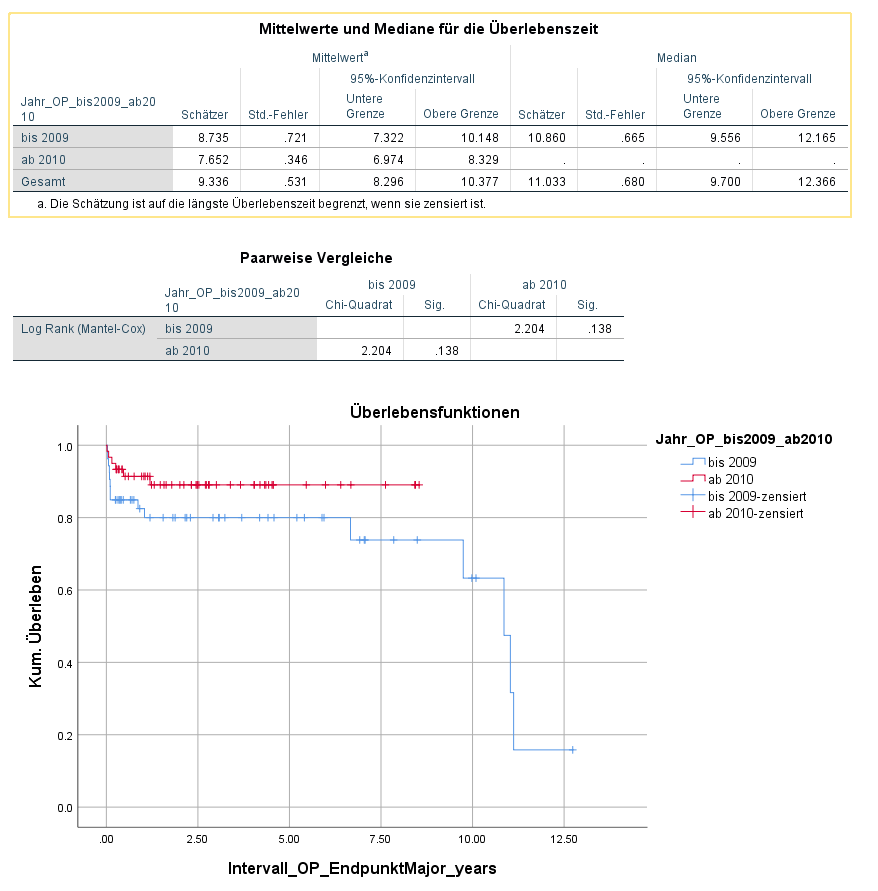


Diabetes yes


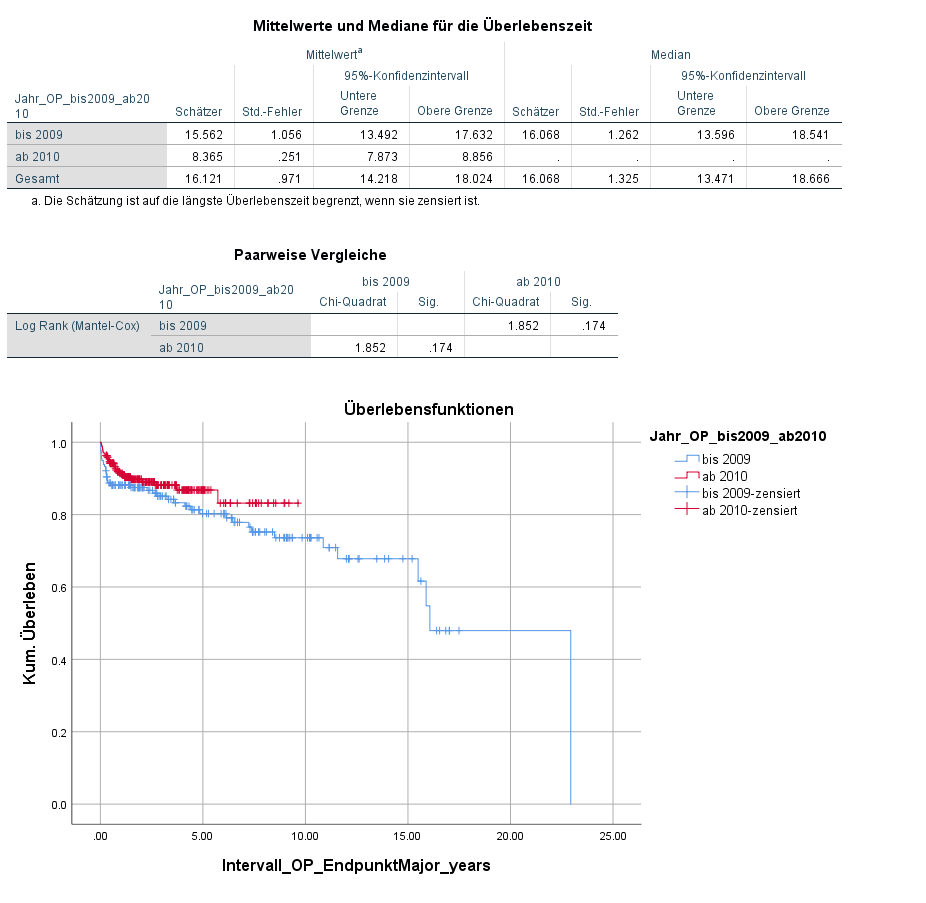


PAD 0-2


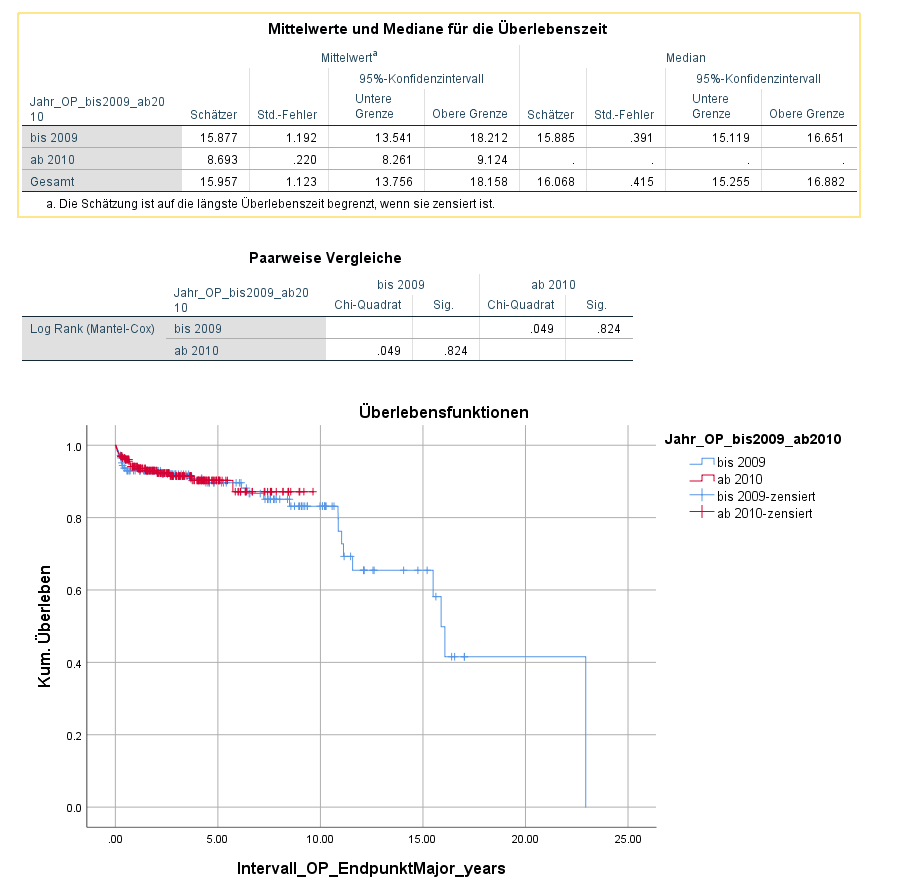


PAD 3-4


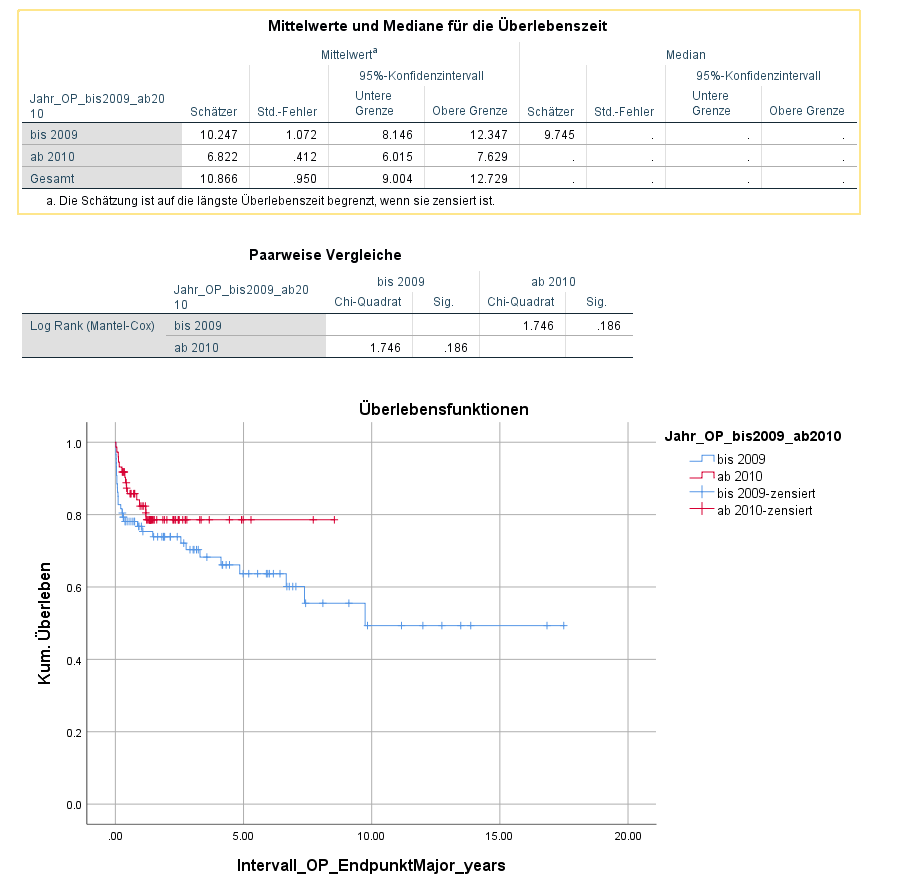

Supplement: Supplementary file 1 — Supplementary file1 (DOCX 492 KB) [file 402_2021_4106_MOESM1_ESM.docx]
